# Acute Pancreatitis and Recurrent Acute Pancreatitis in Children: A 10-Year Retrospective Study

**DOI:** 10.1155/2022/5505484

**Published:** 2022-07-22

**Authors:** Chaohui Wang, Bao Fu, De Su, Ping Huang, Xiaoyun Fu

**Affiliations:** ^1^Department of Critical Medicine, Affiliated Hospital of Zunyi Medical University, Zunyi City, 563000, Guizhou Province, China; ^2^The Severe Acute Pancreatitis Diagnosis and Treatment Center of Guizhou Province, Zunyi City, 563000, Guizhou Province, China

## Abstract

**Aim:**

To compare the clinical characteristics of acute pancreatitis (AP) and recurrent acute pancreatitis (ARP) in children.

**Method:**

From January 2011 to January 2021, a total of 275 pediatric patients with AP admitted to a tertiary teaching hospital were enrolled.

**Results:**

The median age of 275 children was 12.0 years. Among them, 55 cases were ARP. The leading causes of pediatric pancreatitis were biliary tract and virus infection. The percent of male in the AP group was higher than that in the ARP group. Viral infection in the AP group were higher than that in the ARP group, but anatomical abnormalities were lower than those in the ARP group. The incidence of pancreatic pseudocysts in the ARP group was higher than that in the AP group. The median interval time from AP to ARP was 3.0 months.

**Conclusion:**

The main causes of pediatric pancreatitis were biliary tract and virus infection in the study. AP caused by virus infection seems to be less likely to develop into ARP. Female and anatomical abnormality are risks of ARP. Children with ARP are more likely to be complicated with pancreatic pseudocyst. There was no difference in ICU admission or mortality between AP and ARP.

## 1. Introduction

Acute pancreatitis (AP) is a common acute abdomen disease, which is an inflammatory disease characterized by pancreatic edema, bleeding, and necrosis [1]. The clinical features and outcome of adult AP have been widely reported [2]. There are few reports about pediatric pancreatitis, but the incidence is gradually increasing [3]. Currently, our understanding of pediatric AP is still poor.

Little is known about acute recurrent pancreatitis (ARP) in children. The recurrence rate of AP in children is about 13%-35% [4, 5]. ARP has brought a huge burden to children's life, the families, and society. Although some studies have reported the risk factors of ARP in children, the clinical characteristics of AP in children are quite different in vary regions. The aim of this study was to study the epidemiology and clinical characteristics of pediatric AP and ARP in a tertiary teaching hospital in Yunnan-Kweichow Plateau of China. The Yunnan-Guizhou Plateau is a typical karst terrain. Most of the local residents are ethnic minorities and have unique eating habits. Therefore, it has certain clinical significance to understand the clinical characteristics of children with pancreatitis in this area.

## 2. Methods

This is a retrospective cohort analysis conducted in the Affiliated Hospital of Zunyi Medical University. The hospital is a tertiary teaching hospital with 3000 beds. It is also the diagnosis and treatment center of severe acute pancreatitis (SAP) in Guizhou Province of China. This study was approved by the Clinical Trial Ethics Committee of the Affiliated Hospital of Zunyi Medical University. Informed consent was waived.

### 2.1. Study Population

AP in children has been previously defined as the presence of at least 2 of the following 3 criteria: (1) typical clinical symptoms with consistent abdominal pain; (2) serum amylase and/or lipase higher than 3 times the upper limit of normal; and (3) characteristic manifestations of abdominal ultrasound and/or computed tomography [6, 7]. From January 2011 to December 2020, this study enrolled all patients aged <18 years referred to our hospital for a first attack of acute pancreatitis [8]. Pediatric SAP is defined as AP with organ dysfunction lasting more than 48 hours. Persistent organ failure may be single or multiple and may develop beyond the first 48 hours of presentation [9]. ARP is defined by two attacks with at least one month free interval or less than one month if symptoms resolve with complete normalization of enzymes [10]. The etiology of each case of AP and ARP was determined according to previous reports [5, 11, 12]. Magnetic resonance cholangiopancreatography was performed in all children with ARP to detect structural abnormalities of the pancreaticobiliary duct. Serum calcium and lipid analyses were performed in all children with pancreatitis to look for the cause of hypercalcemia and hypertriglyceridemia. Viral infection was diagnosed by serological and/or histological evidence of viral infection [12]. A clinical diagnosis was accepted for a viral infection when classic clinical criteria for diagnosis were met (such as classic parotitis with mumps, Koplik spots and disseminated cephalocaudal rash for measles, or classic zoster/chickenpox rash with varicella zoster virus). A diagnosis of a viral infection with a concurrent diagnosis of AP, a temporal resolution of both entities, and the attempt to exclude the most common etiologies of AP (gallstones, alcohol, medications, and metabolic causes) defined the diagnosis of virus-attributed AP which is congruent with the definition suggested by Makharia et al. [13]. Data collected included laboratory tests, ultrasonic and imaging findings, risk factors, patient management, and interventions, as well as the clinical course and outcomes.

### 2.2. Statistical Analysis

Data were analyzed using SPSS software version 18.0 (IBM Corporation, Armonk, NY, USA). In this study, in the distribution of variables, continuous data were summarized as medians with interquartile range, while categorical data were expressed as frequency counts with percentages. We used Fisher's exact tests to compare the categorical variables between AP and ARP groups. The nonparametric Wilcoxon-Mann-Whitney tests were used to compare the difference of continuous data between the AP and ARP groups. *P* < 0.05 was considered statistically significant for analyses.

## 3. Results

### 3.1. Clinical Characteristics of AP in 275 Children

A total of 275 pediatric patients with first attack AP were enrolled in this study. The median age of 275 children was 12.0 years (IQR: 8.0-16.0). Among them, 13-17 years old accounted for the most (49.8%), followed by 7-12 years old (37.8%) and <7 years old (12.4%). The ratio of male and female is close (49.1% *vs.* 50.9%). Among 275 pediatric pancreatitis, 225 cases were first-onset AP (80.0%) and 55 cases were ARP (20.0%). The top three causes of pediatric pancreatitis were biliary tract infection (37.1%), virus infection (21.5%), and idiopathy (21.1%). 28 (10.2%) pediatric pancreatitis developed into SAP.

The most common symptoms of pediatric pancreatitis are abdominal pain (94.9%), vomiting (60.4%), and nausea (58.2%). The incidence of peritoneal effusion, acute necrotic collection (ANC), and pancreatic pseudocyst (PPC) was 11.6%, 2.9%, and 8.4%. Acute respiratory distress syndrome (ARDS) and acute kidney injury (AKI) occurred in 8 (2.9%) and 7 (2.5%) patients. 23 (8.4%) patients were transferred to the ICU, and 2 (0.7%) children died in the hospital. One child died of severe trauma, and one died of leukemia. The hospitalization time of pediatric pancreatitis was 9.0 days (IQR: 7.0-14.0), and the hospitalization cost was 10163.5 yuan (IQR: 6090.9-23361.1).

### 3.2. Comparison of Clinical Features between AP and ARP

There was no statistical difference in the age of children between the AP group and the ARP group (*P* = 0.05). The proportion of male in the AP group was significantly higher than that in the ARP group (55.9% *vs.* 30.9%, *P* < 0.001, Table 1). In terms of etiology, viral infections in the AP group were higher than those in the ARP group (26.4% *vs.* 1.8%, *P* < 0.001, Table 1), but anatomical abnormalities were lower than those in the ARP group (2.7% *vs.* 12.7%, *P* = 0.006, Table 1). There was no significant difference in the incidence of SAP between the two groups (10.5% *vs.* 9.1%, *P* = 0.77, Table 1).

The onset symptoms of the two groups were similar at onset, and the incidence of vomiting in the AP group was higher than that in the ARP group (65.5% *vs.* 40.0%, *P* < 0.001, Table 1). At admission, the ALT level of the AP group was higher than that in the ARP group (19.0 (11.0-47.5) *vs.* 18.5(12.8-40.5), *P* = 0.04, Table 1). The incidence of pancreatic pseudocysts in the AP group was lower than that in the ARP group (6.4% *vs.*16.4%, *P* = 0.03, Table 1). There was no difference in the outcomes of the two groups.

### 3.3. The Risk Factor of Progression from AP to ARP

We performed a binary logistic regression analysis of the indicators that were different between the two groups, such as anatomical abnormalities, virus infection, pseudocyst, and sex ratio. The results showed that anatomical abnormality (*P* = 0.004; OR = 5.201; 95% CI 1.673–16.174), viral infection (*P* = 0.004; OR = 0.052; 95% CI 0.007–0.382), and sex (*P* = 0.001; OR = 0.359; 95% CI 0.191–0.675) were the independent factors of ARP (Table 2).

### 3.4. Progression from AP to ARP

**Figure 1:**
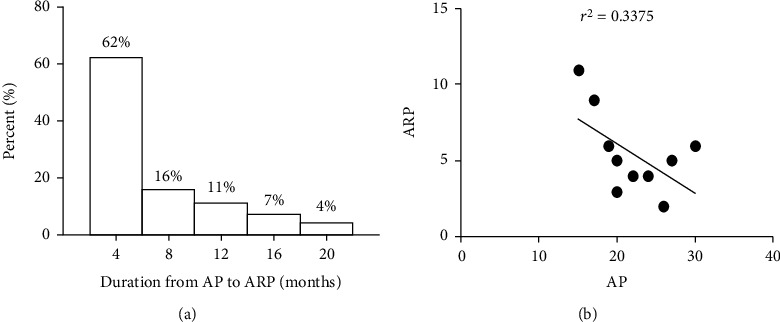
(a) Time to recurrence of pancreatitis from the first episode of AP to second AP. (b) The linear regression analysis on the number of people with AP and ARP every year.

Of the 275 AP patients, 55 (20.0%) progressed to ARP during the study period. The median interval time from AP to ARP was 3.0 months (IQR: 1.5–6.5). The majority, 62%, of the ARP patients developed ARP within 4 months of their first AP attack. The episode distribution of AP progression to ARP is shown in Figure 1(a). We conducted a linear regression analysis on the number of people with AP and ARP every year. The results showed that *r*^2^ = 0.34 and *P* = 0.08 (Figure 1(b)). Therefore, there is no obvious trend change in the incidence of ARP.

## 4. Discussion

In this study, the clinical characteristics and outcomes of AP and ARP in children in Yunnan-Kweichow Plateau of China were analyzed and presented. The etiology of AP in children is significantly different from that in adults. A previous study from the United States found that idiopathy (31%) and drug-related (23%) were the main causes of AP in 115 children [4]. A study involving 130 children with AP showed that biliary tract infection (31.5%), idiopathy (28.5%), and trauma (16.2%) were the main reasons [14]. In our study, the top three causes of pediatric pancreatitis were biliary tract infection (37.1%), virus infection (21.5%), and idiopathy (21.1%). A meta-analysis of 48 studies showed that the top three causes in different continents were gallstones (33%), systemic disease (31%), and infection (29%) in Asia; trauma (32%), idiopathy (25%), systemic disease (16%), and infection (16%) in Oceania; and idiopathy (26%), systemic disease (13%), and infection (13%) in Europe [5]. Therefore, they differed among various continents. In addition to geographic factor, inconsistent inclusion criteria among studies may also account for differences in the etiology of AP.

This study shows that the incidence of SAP in pediatric AP was 10.2%, which is similar to a previous report (12.8%) [4]. The severity of AP in children is lower than that in adults, which may be related to the etiology, complications, and pancreatic necrosis [14]. In this study, the incidence of acute necrotic collection was 2.9%, which was lower than that in a previous study (4.6%) [14]. Raizner et al. reported necrotizing pancreatitis developed in less than 1% of pediatric AP and they suggested that necrotizing pancreatitis in children was associated with severe acute and late complications in children [15]. The incidence of SAP in children varies in different regions. It may have a great relationship with different etiological proportions. In addition, the incidence of other local or systemic complications of acute pancreatitis in children is also low, such as AKI, ARDS, and pancreatic pseudocyst. Although our research and previous literature have reported that the severity of AP in children is lower than that in adults, the underlying pathophysiological mechanism still needs to be further explored.

In this study, 20% of children with AP progressed to ARP. Two previous single-center studies have showed that 10–35% of pediatric AP develop ARP [16, 17]. During the first attack of AP in children, age, maleness, and pancreatic necrosis are related to the progression of ARP [4, 18, 19]. However, female accounted for a high proportion of patients with ARP in our study. Similarly, a recent study showed that 15 of the 19 children in the ARP group were female [14]. This study shows that female is a risk factor for ARP, but large-scale clinical studies are still needed to confirm. In terms of etiology, the incidence of viral infection in the ARP group was lower than that in the AP group in this study. Sweeny et al. also found that viral infection was the lowest risk for progression to ARP over time in their survival analysis [4]. These results indicate that the probability of AP caused by virus infection to progress to ARP is low. Approximately 5-20% of children with AP have anatomical abnormalities in the pancreaticobiliary system [20, 21]. The incidence of anatomical abnormalities was higher in ARP than that in AP in this study. Binary logistic regression analysis showed that anatomical abnormalities was a risk factor of ARP. Therefore, pancreatitis and biliary examinations should be routinely performed in children with ARP.

The incidence of pseudocyst in the ARP group was higher than that in the AP group, while there was no significant difference in other complications. Our results indicate that neither anatomical abnormalities nor pancreatic pseudocysts can predict the occurrence of ARP. There was no difference in outcomes between the AP group and ARP group. Two patients died in the hospital, but none of them died of pancreatitis. The majority of the patients (62%) who developed ARP had progressed within four months. This finding may be helpful for parent consultation and anticipatory guidance for pediatric patients presenting with their first AP attack. The previous studies also showed that the incidence of AP in children has gradually increased in recent years, approaching that of adults [22–24]. In this study, the annual incidence of AP showed an upward trend, but the incidence of ARP did not show an upward trend. This increased incidence of AP may be due to a real increase in morbidity, increased awareness, or increased referrals.

This study has some limitations. First, this study is a single-center study, so the results could not be applied to all children with AP. Second, this is a retrospective study, and etiological studies of ARP-like gene mutation analysis are lacking. Previous studies have showed that genetic causes account for less than 10% of pediatric AP patients, but more than 50% of patients with ARP [3, 22, 25]. Thirdly, our study was conducted in a tertiary teaching hospital, so some mild AP patients may be missed. These patients may be treated in primary hospitals due to their relatively mild condition. Finally, some metabolic disorders can trigger acute or chronic pancreatitis, such as methylmalonic aciduria (MMA), propionic acidemia (PPA), and fatty acid oxidation disorder (FAOD) [26]. However, all patients in this study were not screened for these rare disorders.

## 5. Conclusion

The main causes of pediatric pancreatitis were biliary tract and virus infection in the study. AP caused by virus infection seems to be less likely to develop into ARP. Female and anatomical abnormality are risks of ARP. Children with ARP are more likely to be complicated with pancreatic pseudocyst. There was no difference in ICU admission or mortality between AP and ARP.

## Figures and Tables

**Table 1:** Clinical characteristics and outcomes of AP and ARP.

Variable	AP (*n* = 220)	ARP (*n* = 55)	*P*
Age (years)	11.5 (8.0, 15.0)	12.0 (9.0, 16.0)	0.05
Sex (male)	123 (55.9%)	17 (30.9%)	<0.001
Etiology, *n*%			
Biliary	78 (35.5%)	24 (43.6%)	0.26
Hypertriglyceridemia	6 (2.7%)	2 (3.6%)	0.66
Trauma	14 (6.4%)	4 (7.3%)	0.77
Viral infection	58 (26.4%)	1 (1.8%)	<0.001
Anatomical abnormality	6 (2.7%)	7 (12.7%)	0.006
Drug-induced	6 (2.7%)	3 (5.5%)	0.39
ERCP-induced	3 (1.4%)	0 (0)	0.38
Tumour	2 (0.9%)	0 (0)	0.48
Autoimmune	2 (0.9%)	1 (1.8%)	.49
Idiopathy	45 (20.1%)	13 (23.6)	0.58
Severe AP, *n*%	23 (10.5%)	5 (9.1%)	0.77
Signs and symptoms at admission, *n*%			
Abdominal pain	207 (94.1%)	54 (98.2%)	0.32
Nausea	134 (60.9%)	26 (47.3%)	0.09
Vomiting	144 (65.5%)	22 (40.0%)	<0.001
Abdominal distention	32 (14.5%)	8 (14.5%)	0.99
Fever	16 (7.3%)	2 (3.6%)	0.54
Skin yellowness	3 (1.4%)	0 (0)	0.38
Diarrhea	2 (0.9%)	1 (1.8)	0.49
Laboratory test			
White blood count	10.1 (6.5, 14.7)	8.9 (5.9, 13.4)	0.18
Percentage of neutrophils (%)	78.0 (60.1, 86.0)	74.0 (66.0, 86.0)	0.47
ALT	19.0 (11.0, 47.5)	18.5 (12.8, 40.5)	0.05
Complication, *n*%			
Fluid accumulation in abdomen	26 (11.8%)	6 (10.9%)	0.85
Acute necrotic collection (ANC)	7 (3.2%)	1 (1.8%)	0.59
Pancreatic pseudocyst (PPC)	14 (6.4%)	9 (16.4%)	0.03
Acute respiratory distress syndrome (ARDS)	8 (3.6%)	0 (0)	0.36
Acute kidney injury (AKI)	6 (2.7%)	1 (1.8)	0.70
Outcomes			
ICU admission	20 (9.1%)	3 (5.5%)	0.57
Hospital stay	10.0 (7.0, 14.0)	9.0 (6.0, 14.0)	0.80
Cost	10303.4 (5991.6, 22404.4)	9628.3 (6669.9, 27193.0)	0.67
Mortality	1 (0.5%)	1 (1.8%)	0.36

ERCP: endoscopic retrograde cholangiopancreatography; AP: acute pancreatitis; alanine aminotransferase; ARP: acute recurrent pancreatitis; ANC: acute necrotic collection; PPC: pancreatic pseudocyst; ARDS: acute respiratory distress syndrome; AKI: acute kidney injury.

**Table 2:** The risk of acute recurrent pancreatitis (ARP).

Parameter	OR	95% CI	*P*
Anatomical abnormality	5.201	1.673-16.174	0.004
Viral infection	0.052	0.007–0.382	0.004
Pancreatic pseudocyst	1.673	0.618–4.534	0.31
Sex	0.359	0.191-0.675	0.001

## Data Availability

The raw clinical data used to support the findings of this study are available from the corresponding author upon request.

## Additional Points

*Key Notes*. Compared with adult pancreatitis, we know less about AP and ARP in children. The etiology of pancreatitis in children is complex and has regional differences. We found that the main causes of pediatric pancreatitis were biliary tract and virus infection in Yunnan-Kweichow Plateau of China. AP caused by virus infection seems to be less likely to develop into ARP, while female AP children are more likely to progress to ARP.
